# Using medical storytelling to communicate problems and solutions in the low back pain conundrum: an evidence-based tale of twins

**DOI:** 10.1186/s12998-023-00499-9

**Published:** 2023-08-08

**Authors:** Donald R. Murphy, Brian D. Justice, Jeffrey Borkan

**Affiliations:** 1 Department of Family Medicine, Alpert Medical School of Brown University, 133 Dellwood Road, Cranston, RI 02920 USA; 2 Excellus BlueCross BlueShield, 165 Court Street, Rochester, NY 14647 USA; 3 Department of Family Medicine, Alpert Medical School of Brown University, 111 Brewster St, Pawtucket, RI 02860 USA

**Keywords:** Low back Pain, Neck Pain, Healthcare System, Health System incentive, Healthcare Problem-Solving, Patient-centered care, Relationship-centered care, Medical narratives, Health Care Reform, Primary care, Health Policy, Learning Health Systems, Spine

## Abstract

**Objectives:**

Low back pain (LBP) is the number one cause of disability world-wide. It is also the most expensive area in healthcare. Patient-centered innovations are needed. This paper uses medical storytelling to illustrate the common problems that often lead to unnecessary suffering for patients, and costs to society. We present innovative solutions, including narrative interventions.

**Methods:**

We use medical storytelling to present a scenario in which hypothetical twin patients with identical LBP episodes enter the healthcare system, with one twin managed in an appropriate manner, and the other inappropriately.

**Results:**

One twin becomes a chronic LBP sufferer, while the other experiences quick resolution, despite identical conditions. Recommendations are made to de-implement inappropriate action and to implement a more productive approach.

**Conclusions:**

Many patients with LBP descend into chronic pain. This is rarely inevitable based on clinical factors. Much of chronic LBP results from how the condition is handled within the healthcare system. Medical narrative may be one innovation to illustrate the problem of current LBP management, recommend solutions and foster changes in clinical behavior.

**Practical implications:**

The starkly different outcomes for each identical twin are illustrated. Recommendations are made for reframing the situation to de-implement the inappropriate and to implement a more appropriate approach.

## Background

Over the past few decades low back pain (LBP) has become both the most common cause of disability world-wide [1] and the most expensive area of healthcare in the US [2], despite national and international efforts. The problem is prevalent throughout the Western world [3]. Transition from acute to chronic LBP is particularly problematic, and accurately predicting who will progress remains elusive. 10% of cases become chronic, causing great personal suffering [4] and expense, accounting for 75% of the total cost of LBP [5, 6]. In most LBP episodes, there is no inherent factor that inevitably leads to chronic pain and disability. Rather, the likely biggest factor is the manner in which the episode is handled when a patient enters the healthcare system [7].

Recent evidence suggests that contextual factors can play an important role in outcomes of care [8]. Contextual factors can be influenced by the style and content of communication [9] as well as clinical beliefs [8] and behaviors [8] on the part of healthcare practitioners. In addition, action steps in diagnosis and management can negatively (e.g. inappropriate imaging [10], unnecessary opioid prescription [11]) or positively (e.g., advice to stay active [12], promoting therapeutic alliance [13]) influence outcome of care [10, 14, 15]. In the area of LBP, and in the healthcare system at large, de-implementing inappropriate health interventions is increasingly recognized as a priority [16].

Attempts have been made over the past few decades to raise awareness and bring systematic changes toward greater efficiency in the management of patients with LBP. This has come about through editorials and commentaries [3, 17, 18], guidelines [19, 20] consensus documents [21–23] and entire journal issues dedicated to the topic [24, 25]. Changes are occurring [26] but continued efforts are needed to illustrate the problems with current approaches to LBP and solutions that are needed. Primary-level clinicians, such as chiropractors and physical therapists, particularly those serving in the role of primary spine practitioner [26], are increasingly seen as a key to bringing about systematic improvements in the care of patients with LBP. However, we feel that it is important for these practitioners to also play a role in communicating to others the problems and potential solutions to the present LBP conundrum [3].

One approach that has been useful in understanding, communicating, and treating patients has been medical storytelling or medical narratives. Narrative methods have been utilized to great effect in the past [27, 28], including in LBP research [29, 30]. Medical narratives have the potential to help both medical and non-medical individuals make sense of complex events while providing meaningful insights into disease, illness, suffering, and the nature of healing. Narratives may also complement quantitative or empirical research, education and practice through their integrative, expressive nature. Finally, there are narrative approaches to treatment in which a practitioner may help influence the trajectory of a patient’s story.

Medical narratives can involve a practitioner in relation to a patient, a practitioner in relation to him- or herself, a practitioner in relation to colleagues or a practitioner in relation to others in the healthcare system [31]. As discussed by Charon [32] there are several examples of the use of each of these approaches. Charon presents a narrative in which we hear a patient’s experience of a medical illness (in that case, the mother’s experience of her child’s illness). Borkan [33] uses medical narrative as a way to present a clinical case in a way that illustrates the non-medical as well as the medical aspect of a physician’s experience. A narrative can be expressed in the first person or the third person depending on the context [33] and for our purpose we are using the third person. Our paper utilizes composite medical narratives based on multiple patients we have seen over the years for the purpose of highlighting certain narrative elements and LBP disease/illness experiences. Though it involves implicit and explicit bias in selecting or deselecting of narrative elements, it also facilitates elucidating underlying themes and teaching points that cannot be accomplished with a single patient. It also has the added ethical benefits of maintaining anonymity [34].

We present our narrative in the two ways described by Ricouer [35]. In cosmological time - a linear succession in which the chronological “river of time” passes from point A to point B to point C – and as phenomenological time – the experience of present events in relation to the past and future. The construction of our narrative is influenced by our collective experience, in phenomenological terms, of the many similar patients we have seen over the years. And our narrative as well as our recommendations are informed by our understanding of the scientific literature in the field, indicated in our “Strength of Recommendation”. The strength of recommendations is based on the best available current evidence rather than depending purely on our personal experience. The use of a composite, rather than an individual patient, helps us illustrate the important messages we are trying to convey. But it is important to recognize the importance of, in a clinical environment, seeing each patient as a unique individual.

The primary purpose of this paper is to use medical narrative to illustrate to clinicians, policymakers, academicians, and others who may not have expertise in efficient management of LBP, to help them recognize important clinical action steps and decision points that can lead to chronic pain and disability, or to better patient outcomes. Secondarily, we present the importance of medical narrative in helping practitioners understand each patient’s story, and to utilize that in taking a respectful, empathetic and nourishing approach to care [32]. We present the narratives in the third person as a convenience, though it could be relayed in the first or second person.

We follow identical twins with identical episodes of LBP, whose journeys into the healthcare system start from the same point. The twins go down two very different clinical paths, leading to remarkably different outcomes. These case histories represent two very common scenarios that the authors (and many others) have encountered over the course of many years. We provide best evidence to assess the quality of clinical decision making at each juncture.

## Methodology

The LBP narratives utilized here are composites based on patients treated by the authors in their primary care and/or referral spine clinics. The narratives were jointly constructed and do not represent any single patient, set of twins, or occurrence.

**Fig. 1:**
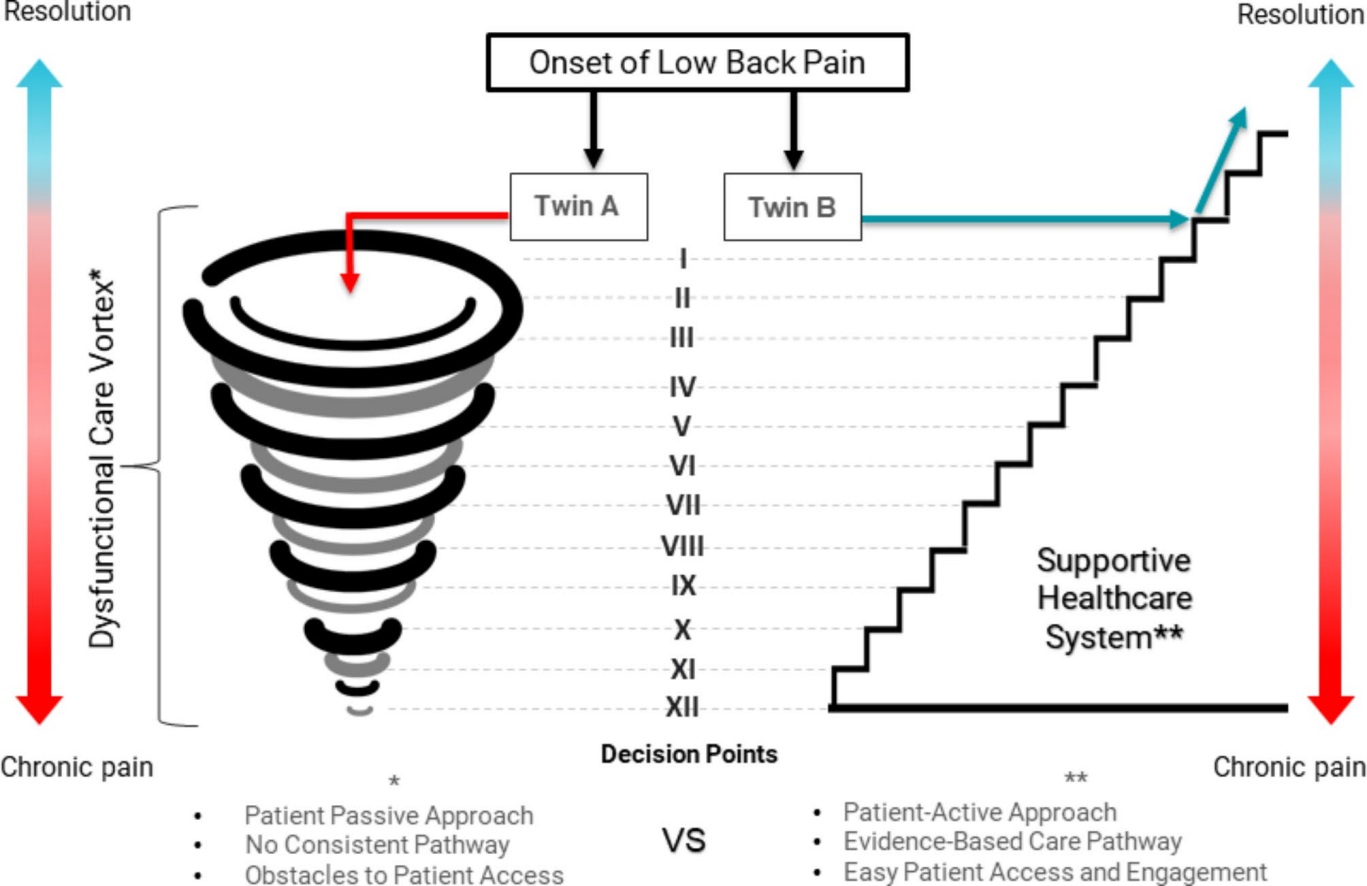
Illustration of the course of each twin

Periodically throughout the case history of Twin A, “Decision Points” are identified with Roman numerals, each of which is discussed in Table 1 and illustrated in Fig. 1. Evidence to support the “Decision Points” for care of LBP patients is based on “best evidence”. This evidence has been gleaned from systematic reviews and randomized controlled trials (A), inconsistent or limited quality patient-oriented evidence (B), or consensus, usual practice, expert opinion, disease-oriented evidence, and case series (C) [36].

### Narratives

#### Twin A

An otherwise healthy 47-year-old man presents to his primary care practitioner (PCP) with LBP which started at home two days earlier. The patient states that he and his identical twin brother both bent forward to lift an old footlocker filled with family memorabilia and simultaneously felt sudden pain in their lower backs.

Twin A describes the pain as “8 out of 10”. It is well-localized to the lumbosacral area without radiation into the lower extremities. The pain is provoked by movement and is relieved by rest. He denies abdominal symptoms, weakness, paresthesia, numbness, fever, chills and constitutional symptoms.

On examination, the patient is sitting uncomfortably on the examination table. Lumbar range of motion is reduced, and straight leg raise provokes the LBP bilaterally but there is no radiation of pain. Sensory, motor and reflex examinations are unremarkable.

The patient is diagnosed with “non-specific low back pain” and plain radiographs are ordered^1^. They reveal degenerative disc disease at L3-4 and L4-5. The patient is told by his PCP that he has two degenerative discs likely causing his low back pain^1^. Non-steroidal anti-inflammatory drugs are prescribed. The patient is told to stay out of work to prevent worsening of the problem and that he should avoid activities that provoke pain^II^. The patient is scheduled to follow up in two weeks.

At two-week follow up, Twin A states that the pain has not changed. Physical therapy is recommended. The patient asks, “How can physical therapy help if the discs are already degenerated?”^I^ It is explained that referral for physical therapy is the standard protocol prior to considering seeing a surgeon, and that the insurance carrier will not pay for the surgeon visit until after he has tried physical therapy^III^. The patient reluctantly agrees to physical therapy. It is recommended that in the meantime he should continue with the medications and should remain out of work^II^. The patient sees a physical therapist, who recommends exercise. The patient is concerned about doing exercises, feeling that he must remind the physical therapist that “I have two discs that are degenerating.” He reluctantly follows the recommendation but stops because he feels that the exercise is causing discomfort. He thinks to himself, “it is aggravating my degenerating discs”. He considers discussing this with the physical therapist, but he decides that it does not make sense for him to continue physical therapy anyway, because he must pay a substantial copayment on each visit, and it would be cheaper to just see a surgeon^IV^, which he can do now since, in his mind, he can make the point that physical therapy has “failed”.

Twin A stops physical therapy^V^ and returns to his PCP, requesting an MRI to “figure out what’s going on”. MRI is ordered, which confirms the presence of degenerative disc disease at L3-4 and L4-5, and additionally reports facet arthrosis at those levels as well as disc bulges at L3-4, L4-5 and L5-S1^VI^. The patient receives an automated email message that the MRI report is available^VII^. He quickly signs into his electronic health record portal, reads the report and immediately calls his PCP in a panic, worried about the wording he reads on the report. His PCP confirms the findings of the MRI and refers him to a spine surgeon^VIII^. It is recommended that the patient remain out of work until he sees the surgeon^II^. The first available appointment with the surgeon is scheduled in six weeks^IX^. In the meantime, the patient remains at home, being careful to avoid any activity he thinks will aggravate his condition and becomes increasingly worried about the prospect of having to have surgery on this back. He tries to figure out how to plan for his future given that, in his mind, it is likely he will never be able work again and worries about how he will be able to support his family while coping with his disability.

Finally, he sees the spine surgeon, reporting that his pain has been steadily worsening. The surgeon reviews the MRI with him, confirming the multi-level degenerative changes and explaining that he may need fusion surgery, but that the standard protocol is to try injections first^X^. The surgeon tells the patient to remain out of work^II^ and refers the patient to an interventional physician, whom the patient sees at the first available appointment, one month^IX^ after the surgeon visit. A series of three injections is recommended, each occurring one-month apart^XI^. The patient is told to remain out of work.^II^.

Twin A undergoes the three injections and follows up with the surgeon, four months^IX^ after his initial visit. He tells the surgeon that his pain has not changed and that, if anything, it seems to be worsening. He also explains that he is very upset with his PCP because of the delay in “getting the MRI to clarify the situation” and getting him on the injection regimen sooner, when, in his mind, it was more likely to be beneficial. It is explained to the patient that he has two choices: have surgery to attempt to correct the problem or “learn to live with it”^XII^. The patient discusses the situation with his wife and decides that “I can’t continue living like this; I have to get this fixed”. He agrees to the surgery.

We encounter the patient six months later. He is taking opioid medications “just to get through the day”. He had been certified as permanently disabled and is living on disability payments which “barely allow me to survive and support my family”. He is despondent, saying that “I used to be a young, strong, productive man who was able to support his family. Now look at me”. He is filled with anger at his PCP (“who didn’t act fast enough in getting this fixed”), the physical therapist (“who tried to force me to do exercises despite the fact that it made the degenerating discs worse”), the interventionist (“who strung me along with those useless injections”), the surgeon (“who must have botched the operation”) and the insurance carrier (“who made me waste time and money on useless physical therapy”).

#### Twin B

An otherwise healthy 47-year-old man presents to his PCP with LBP which started when lifting the footlocker with his brother one day earlier. History and exam are exactly the same as with Twin A, except that the PCP applies manual palpation in the lumbosacral area that exactly reproduces the pain [37–39]. The patient says, “that’s it, you found the pain! What is it?” [40].

Twin B is told that he has a “mechanical” problem and that there is no indication of a “nerve” problem, a severe injury or serious medical disease. It is explained that this is good news because, although “mechanical” pain can be intense at times, it almost always gets better, and there is much that can be done to quickly move the recovery along. It is explained that the best thing is to be as active as possible and to do some simple exercises. The option is offered to refer him to a doctor trained in evidence-based, patient-centered, primary-level spine care [26, 41–43] who can give him more specific information and can get him on a strategy targeted at his particular problem. The patient takes that option. On checking with his insurance carrier, he learns that there is minimal out-of-pocket cost for pursuing active, noninvasive, guideline-concordant care.

Over the following few weeks, the patient is managed with an approach that is primarily focused on self-care, in which he is taught specific exercises, based on a detailed history and exam, that he says, “hurt a bit at first, but really helped”. He is also guided on a process of gradually increasing activities, that he felt “got me ready to get back to work”.

After three weeks of working on a light duty basis, Twin B is back to work full duty and is engaging in normal activities at home. He says that he has occasional discomfort but feels confident in his ability to self-manage any recurrence that arises, based on what he has been taught.

## Discussion and conclusion

### Discussion

The dramatically different outcomes for these twins cannot be explained based on the actual condition in each case, as the brothers, and their conditions, were identical. The difference between these two brothers’ situations lies primarily in the response within the healthcare system and the collaborative engagement of the patients and their providers.

In presenting the case of Twin A, several “Decision Points” are demarcated in which something impactful occurred, which helped lead the patient down the road toward chronic pain. At each of these points, a different choice could have been made, which likely would have helped redirect the patient toward resolution. In Table 1, we look at each Decision Point, identify why the action taken in each case was problematic, what alternative could have been made, and how the alternative could have helped lead to a very different outcome.

**Table 1:** A discussion of each Decision Point, the impact of each on the patient’s story, and how the story could have followed a different path

Decision Point	What went wrong?	How did this contribute to chronicity?	What could have been done differently?	What would have been the likely result?	How might that help the patient’s story be more like that of Twin B?	Strength of Recommendation [36]
I	“Radiographs are ordered” without indication [19] and the “patient is told he has two degenerative discs causing his low back pain”	“Disc degeneration” is found, and this is presumed the cause of the pain, resulting in unfounded fear and catastrophizing [10, 44, 45]	No radiographs ordered [19]	The graphic, fear-inducing idea of having a “degenerating disc” would not have entered the patient’s mind	“My back hurts a lot but that does not mean it is damaged.”	A Based on consistent and good quality patient-oriented evidence
II	“told to stay out of work to prevent worsening of the problem and that he should avoid activities that provoke the pain”	The patient is instructed to take a passive approach, and to believe that activities should be avoided	A defined plan to resume activity is discussed and progressively return to all usual activities, including work [20, 46]	Hastens recovery and early return to work [47]	“Even though my back hurts, I can engage in activity. In fact, activity will likely help.”	A Based on consistent and good quality patient-oriented evidence
III	“.referral for physical therapy is the standard protocol prior to considering seeing a surgeon, and…the insurance carrier will not pay for the surgeon visit until after he has tried physical therapy”	The patient feels that the conversation about referral for physical therapy served the purpose of following a protocol, rather than listening to him and framing the referral on his needs in overcoming the problem [29]. And that seeing a surgeon is inevitable, and necessary; being referred for physical therapy is merely a formality [48, 49]	After listening carefully to the patient’s concerns, a clear plan to rapidly bring about resolution of the problem, focusing on a targeted, evidence-based approach best suited to his condition	Rapid resolution of the problem [50]	“They are all on the same page in helping me get better as quickly as possible.”	A Based on consistent and good quality patient-oriented evidence
IV	The patient terminates physical therapy “because he has to pay a copayment on each visit, and it would be cheaper to just see a surgeon”	The patient is disincentivized to pursue appropriate care for his condition [20, 51] because his insurance company policy puts a financial barrier in place that make appropriate care substantially more costly to him then inappropriate care [52]	A policy in place that, at all levels of the healthcare system, puts incentives in place that encourages high-value care and discourages low-value care [52]	No barrier is in place for the patient to pursue the most appropriate treatment	“The exercise hurts a bit but I might as well stick with it – there’s no reason not to.”	B Recommendation based on inconsistent or limited quality patient-oriented evidence
V	“He stops physical therapy”	In terminating physical therapy, he loses the opportunity to gain an understanding of the discomfort he experienced when exercising [53]. The perception of his having a “degenerating spine” is reinforced, as is the assumption that activity should be avoided in order to prevent further “damage” [54]	A policy in place that incentivizes patients to pursue active, evidence-based, patient-centered care	The patient has an opportunity to be educated regarding the concept of “hurt ≠ harm”, leading him to continue an evidence-based approach [55]	“At first I was a little worried when the exercise bothered my back. But the doctor assured me that ‘hurt does not necessarily mean harm’ and, sure enough, they were right.”	A Based on consistent and good quality patient-oriented evidence
VI	“MRI is ordered which confirms …degenerative disc disease… facet arthrosis… disc bulges”	Another inappropriate imaging study [56] reinforces in the patient’s mind that he has a degenerating spine; in fact, his perception is that the MRI showed that his spine is even more severely “damaged” than previously thought [57, 58]	No MRI is ordered, after the practitioner listens carefully to the patient’s concerns. The practitioner provides an individualized explanation as to why MRI is unnecessary; the patient receives evidence-based information about his condition [59, 60]	Reinforcement of the concept that he does not have a fragile spine and that there is no “damage” of concern [59]	“The doctors found the problem on examination and explained it to me. So I don’t have to take the extra time and effort to go for an MRI.”	A Based on consistent and good quality patient-oriented evidence
VII	“The patient receives an automated message that the MRI report is available”	The patient sees confusing, fear-invoking words on the MRI that is provided to him on a piece of paper, without the benefit of expert explanation, context and guidance. This further exacerbates the fear and catastrophizing already in place [44, 58, 61, 62]	[If it were a situation in which an MRI had been ordered] Evidence-based explanation of the report, given verbally and/or imbedded within the report [63], with assurance that all the findings are age-appropriate, dynamic, and very common in patients of his age who have no back pain [44]	Enables the patient to question his own perception of having a “degenerating spine”, opening the door to reframing his situation in a more realistic, accurate and empowering manner [64]	“I have been given information about my back that makes me feel a lot better about what the pain means and what it doesn’t mean.”	B Recommendation based on inconsistent or limited quality patient-oriented evidence
VIII	“His PCP confirms the findings of the MRI and refers him to a spine surgeon”	The patient’s distress that arose from having seen the MRI report is reinforced and exacerbated by being told he has to see a surgeon [65, 66]	In addition to the evidence-based explanation discussed above, assurance that there is no indication that an operation is necessary	Further supports the patient in questioning his perception of a “degenerating spine”, opening the door to an understanding that a straightforward, non-invasive solution is likely to be successful [64]	“The doctors understand me and my back pain, and have pointed me in the right direction.”	B Recommendation based on inconsistent or limited quality patient-oriented evidence
IX	“The first available appointment with the surgeon is scheduled in six weeks”	The patient is left to stay at home and agonize over his predicament, reinforcing both his perceptions of pain and disability, rather than engaging in active steps toward improvement	The patient is immediately directed toward an active, productive approach, with return to activity and work (even if limited)	Avoids the detrimental impact of passivity [67]	“I have been given a bunch of simple things to do for myself. So I don’t have to wait for someone else to do things for me.”	C Recommendation based on consensus, usual practice, expert opinion
X	“The surgeon reviews the MRI with him, confirming the multi-level degenerative changes and explaining that he may need fusion surgery, but that the standard protocol is to try injections first”	Two invasive approaches are discussed without sound evidence supporting their role for his condition [68]	Educating the patient regarding the benign nature of the MRI findings, assuring him that no invasive interventions are needed and indicating that active strategies, founded in self-care, is the best option [66, 69]	Reframes the patient’s impression of a “degenerating spine” that needs passive, invasive treatments	“At first my back hurt so much, I wondered if I would end up needing an operation. I am so relieved that they helped me understand what was going on and get me on the right path.”	A Based on consistent and good quality patient-oriented evidence
XI	“A series of three injections is recommended, each occurring one month apart”	A non-evidence-supported approach is recommended (a “series of three”) [70] that further prolongs the patient’s period of disability and passivity, further leading him on the downward spiral toward chronic pain	Educating the patient regarding the benign nature of the MRI findings, assuring him that no invasive interventions are needed and indicating that active strategies, founded in self-care, is the best option [66, 69]	Reframes his unrealistic perception of his condition and redirects him toward an active approach to the problem	“I was surprised that back pain like this could be get better so quickly and simply.”	A Based on consistent and good quality patient-oriented evidence
XII	“It is explained to the patient that he has two choices: have surgery to attempt to correct the problem or ‘learn to live with it’”	Reinforces his despondency and his inaccurate (though understandable) notion that he has no control over the condition, and that the only hope for him is a passive, invasive, non-evidence-based approach [71]	Educating the patient regarding the benign and dynamic nature of the MRI findings, assuring him that no invasive interventions are needed and pointing out that active strategies, founded in self-care, are the best option [66, 69]	Reframes his inaccurate perception of his condition and redirects him toward an active approach to the problem	By this point, the patient still has a “back pain story”, but only as a memory, and as a useful tool. He can use his back pain story as a reminder, if he has a recurrence of back pain (which many people do), of the helpful approach that was taken, the speed with which he recovered, and the benefit of activity in the recovery. In addition, he can tell his story to others who find themselves in a similar back pain situation as encouragement, and as useful information regarding what worked so well for him.	A Based on consistent and good quality patient-oriented evidence

The hypothetical scenario in which Twin A descended into chronic pain and disability was avoidable, illustrated by the starkly different path of his twin brother, who quickly and successfully overcame the problem. Twin A’s path resulted from specific, identifiable factors that warrant re-examination so this common scenario can be avoided.

Widespread changes in attitudes and approaches are required to avert a repetition of Twin A’s trajectory in the multitude of LBP sufferers. The first necessary change relates to the way LBP patients are managed, both in communication and in action. This requires all professionals a patient encounters - medical, administrative and otherwise – to be familiar with the current understanding of the etiology, diagnosis and management of LBP, and to be “on the same page” regarding the realities and mythology around this topic [72].

Second, we must establish systems of care in which patients routinely see “the right practitioner at the right time at the right place” and in which obstacles to patients receiving the highest-value care possible are removed. One recent effort in this regard has been the establishment of formalized clinical pathways in which various professionals are put in place, with each having a defined role and who function as a team in the management of communities of patients with LBP [73]. A clinical pathway has been defined as “a complex intervention for mutual (between patient and clinician) decision-making and organization of care processes for a well-defined group of patients during a well-defined period” in which there is clear communication between all team members, and between the team and its patients [74, 75]. A well-functioning clinical pathway is one that is organized in a way that enables each patient to receive the professional service(s) he or she needs and not to receive unnecessary or inappropriate services. Important is the recognition of whether a particular individual with LBP needs to become a “patient” at all [76]. One recent approach is based on the concept of “primary spine care”, in which a well-trained primary-level professional functions as the first contact, and who has the knowledge and skills to management the majority of patients without the need for specialty investigation or intervention [26, 76, 77]. A challenge with this concept is that it necessitates the “primary care” role to be played by a practitioner type other than the traditional primary care practitioner, due to the time and training constraints on that professional group [78, 79]. Given the fact that chiropractors and physical therapists may be the most appropriate professionals to be “retooled” to play the “Primary Spine Practitioner” role, negotiation of political issues may be required in many jurisdictions and countries, and open-mindedness to unconventional innovative approaches to making necessary changes within the healthcare systems.

Finally, we must remain cognizant of the powerful influence language, patient education, motivation and early care have on a healthful or hurtful outcome. In addition, there is the opportunity to work with Twin A to modify his narrative from one of suffering and disappointment to a focus on function rather than pain, resilience rather than vulnerability, and capacity building rather than disability. This can be done by first listening to the patient’s narrative, ideally without disruption, and then discussing options for narrative modification. This might take the form of asking about the most meaningful aspects of the patient’s life, such as raising his children or finding fulfillment in his work roles and seeing how he might engage in these activities even with some level of back pain. Additional opportunities for modifying the patient’s story arise from the interpretation of lumbar disc “abnormalities”. Here too there is a choice of narratives. For example, working with the patient to understand that discs are living tissues and that clinically relevant disc abnormalities can often improve over time, rather than being static over a lifetime. Similarly, the practitioner might help the patient switch from a “rest with back pain” story or plot line to a “stay as active as possible” one. Such narrative coaching approaches may require new skills and competencies on the part of the practitioner. Challenging, but important, is to tailor communication to things that are of greatest value *to the patient*, including life goals and meaningful activities as well as community and identity values. Resources are available for practitioners to hone their skill in utilizing medical narratives in the context of patient care [80, 81].

Helping patients experience a story that is more akin to that of Twin B than of Twin A not only requires clinicians who “do the right thing” (and not the wrong thing). An empowered patient is also key to success in overcoming LBP. Important in empowering a patient is taking an approach from the very beginning that is focused on self-management [82]. The simple act of providing exercises or self-treatment strategies from the beginning is an effective way to not only reduce pain intensity, but to allow the patient to have their first experience of benefit come from something they did to themselves, rather than something that someone else did to them. This helps build self-efficacy [83] and sets the stage for the patient to decide for themselves (with guidance) that they are more capable than they may have assumed before, and that the key to recovery is active engagement and movement, as opposed to avoidance and rest. This can allow a natural segway (again, with guidance) to the patient taking charge of the problem in other ways as well, such assertiveness in decision making, resource utilization and self-monitoring [82].

Rita Charon, a leader in narrative medicine, notes that, “Along with scientific ability, physicians need the ability to listen to the narratives of the patient, grasp and honor their meanings, and be moved to act on the patient’s behalf. This is narrative competence, that is, the competence that human beings use to absorb, interpret, and respond to stories” [32]. This case is an exemplary one where narrative work, along with other treatment interventions, can truly make a difference in the patient’s trajectory.

### Conclusion

Medical storytelling is a method by which important concepts can be communicated that illustrates an important problem and proposes solutions. The identical twins in this story, who had an identical mechanism of back injury, ended up going down very different paths, with startlingly different outcomes. The patients are composites, yet many individual patients have experiences like Twin A. We think that in the vast majority of cases, Twin A’s situation is avoidable. Twin A’s descent into chronic pain and disability resulted from system-driven behavior that pervades his entire healthcare experience. All of it was likely preventable and correctable. This necessitates systematic de-implementation of the inappropriate application of communications, interventions and policies, and the implementation of a more productive, empowering and strategic approach to the management of patients with LBP [76]. Taking various approaches to communicating the issues related to the present LBP conundrum [3], including medical storytelling, can help primary spine practitioners [26] and others to facilitate necessary changes.

### Practical implications

Medical storytelling can serve the purpose of improving patient care in two ways. First, within the practitioner-patient relationship a practitioner, by attuning to the patient’s story, gain a greater understanding of the patient, establish therapeutic alliance and guide a patient to consider alternative aspects of the story that helps lead them toward resolution of the problem.

Second, medical storytelling can be used as a tool to improve patient care systemically. The narratives presented here demonstrate the stark contrasts between the “productive” way and the “non-productive” way that LBP cases are handled as a teaching tool in bringing attention to needed changes in the management of LBP patients, understanding that most LBP patients’ journeys hover somewhere between these two scenarios. Systemic changes are needed toward a best evidence, patient active, pathway-driven approach to healthcare that is applied and reinforced by providers, payers, employers and individuals. Guidelines have been published that provide some direction [19, 20, 84–86], and steps have begun in some communities [77, 87]. Further evolution requires concerted efforts among all stakeholders. More work is required at each level of care, in the coordination of LBP services, and in the positive collaboration and engagement of patients. We are in the midst of an epidemic of chronic LBP, much of which is system driven. Appropriate education of patients and professionals, coordination of services and well-directed incentives are necessary achieve higher levels of health and function.

## Data Availability

Not applicable.

## Abbreviations

LBP: Low back pain
PCP: Primary care practitioner
